# Conventional and Unconventional Use of Lasers in Skin Disorders

**DOI:** 10.1155/2015/934931

**Published:** 2015-03-09

**Authors:** Silvia Moretti, Michael S. Kaminer, Anne Le Pillouer-Prost, Piero Campolmi

**Affiliations:** ^1^Section of Dermatology, Department of Surgery and Translational Medicine, University of Florence, Ospedale Piero Palagi, Viale Michelangelo 41, 50125 Florence, Italy; ^2^SkinCare Physicians, Inc., 1244 Boylston Street, Chestnut Hill, MA, USA; ^3^Yale School of Medicine, New Haven, CT 06510, USA; ^4^Hôpital Prive Clairval, 317 boulevard du Redon, 13009 Marseille, France; ^5^Dermatology Clinic, Department of Surgery and Translational Medicine, University of Florence, Ospedale Piero Palagi, Viale Michelangelo 41, 50125 Florence, Italy

Over the last 50 years, laser sources have been used in many skin diseases, and their role has been increasing in most fields of dermatology. At present, laser technology allows us to treat a variety of skin conditions, either inflammatory or neoplastic, in addition to aesthetic problems.

Hence, in the last few years, surgical, vascular, selective, and other lasers have become more popular. A significant advantage of lasers is their ability to provide “noncontact surgery,” since lesion vaporization occurs without any physical contact between skin and the device. This assures sterilization and helps avoid possible infections.

The different types of laser treatments have been expanding as well as laser technology.

A better understanding of interactions between laser radiation and tissues has made lasers more powerful and selective for each type of lesion and aesthetic problem. Due to the specific properties of selective photothermolysis, lasers exert effects strictly limited to the treated area, avoiding any damage to the surrounding skin. This unique attribute of lasers allows physicians to treat even very small skin lesions with excellent aesthetic results.

In particular, in the vascular field, dye lasers have become the gold standard in treating superficial vascular disease and are excellent for treating large lesions such as port wine stains. Pulsed dye lasers are also useful tools for the so-called unconventional treatments, such as hypertrophic scars, keloids, superficial basal cell carcinoma, and common warts. ND-Yag lasers are used for treating deep vascular lesions and hair removal. In addition, these two lasers can also be employed effectively in combination on difficult lesions, as reported by K. Vas et al., showing that sequential combined treatment with both dye and ND-Yag lasers is satisfactory for surgical scars.

Another popular device is the Q-switched laser, which is effective and safe for removal of benign pigmented lesions and tattoos since it interacts with the dermal structures holding exogenous pigment.

Tattoo removal may be associated with a number of cutaneous side effects, such as inflammatory or rare neoplastic skin reactions, which are described by A. Bassi et al. They highlight that early diagnosis is essential for proper treatment. Intense pulsed light is a versatile tool suitable for many different skin conditions thanks to its wide range of wavelengths, and unconventional uses are exhaustively discussed by D. Piccolo et al.

The constant improvement in laser devices and technology results in the need for continuous training and evolving knowledge. For example, A. Soriani et al. highlight this concept with a study that examines the power loss of laser beams induced by various types of tissue. Since laser penetration is closely related to laser settings such as wavelength and spot diameter, they evaluate these variables in order to accurately determine the fraction of released energy in order to obtain better clinical results.

Due to the increasing need for noninvasive technology for diagnosis, therapy, and follow-up, high resolution tools such as nonlinear optical microscopy and two-photon fluorescence microscopy are in depth presented by R. Cicchi et al. They evaluate possibilities to monitor the effect of therapy and topical absorption.

Since the 1970s, photodynamic therapy aroused a growing interest in treating several skin diseases. E. Filonenko et al. evaluate the effectiveness of this treatment for penile in situ carcinoma, obtaining satisfactory oncological results without affecting patient quality of life. In addition, X. Wang et al. emphasize that these therapies may require long application times so that the use of an automatic medical manipulator system can increase the radiation uniformity and ensure a better therapeutic result.

Due to the growing importance of laser techniques in dermatologic practice, ongoing multicenter studies will be valuable in helping us develop precise standardization of parameters used for each laser and each disease. This will improve the effectiveness of the tools and allow physicians to obtain better and more reproducible results.

*Silvia Moretti* *Michael S. Kaminer* *Anne Le Pillouer-Prost* *Piero Campolmi*

